# In situ synthesis of hierarchically-assembled three-dimensional ZnS nanostructures and 3D printed visualization

**DOI:** 10.1038/s41598-022-21297-y

**Published:** 2022-10-10

**Authors:** Taehwan Lim, Seung Kwon Seol, Hyo-Jeong Kim, Yang Hoon Huh, Yeonwoong Jung, Hee-Suk Chung, Jung Han Kim

**Affiliations:** 1 Advanced Textile R&D Department, Korea Institute of Industrial Technology, Ansan, Gyeonggi-do 15588 South Korea; 2 Smart 3D Printing Research Team, Korea Electrotechnology Research Institute, Changwon, 51543 South Korea; 3 Electrical Functionality Material Engineering, University of Science and Technology (UST), Changwon, 51543 South Korea; 4 Electron Microscopy Research Center, Korea Basic Science Institute, Ochang, 28119 South Korea; 5 NanoScience Technology Center, University of Central Florida, Orlando, FL 32826 USA; 6 Department of Electrical and Computer Engineering, University of Central Florida, Orlando, FL 32816 USA; 7 Department of Materials Science and Engineering, University of Central Florida, Orlando, FL 32826 USA; 8 Analytical Research Division, Korea Basic Science Institute, Jeonju, Jeollabuk-do 54907 South Korea; 9 Department of Materials Science and Engineering, Dong-A University, Busan, 49315 South Korea

**Keywords:** Materials science, Nanoscience and technology

## Abstract

Nanomaterials have gained enormous interest in improving the performance of energy harvest systems, biomedical devices, and high-strength composites. Many studies were performed fabricating more elaborate and heterogeneous nanostructures then the structures were characterized using TEM tomographic images, upgrading the fabrication technique. Despite the effort, intricate fabrication process, agglomeration characteristic, and non-uniform output were still limited to presenting the 3D panoramic views straightforwardly. Here we suggested in situ synthesis method to prepare complex and hierarchically-assembled nanostructures that consisted of ZnS nanowire core and nanoparticles under Ag_2_S catalyst. We demonstrated that the vaporized Zn and S were solidified in different shapes of nanostructures with the temperatures solely. To our knowledge, this is the first demonstration of synthesizing heterogeneous nanostructures, consisting of a nanowire from the vapor–liquid–solid and then nanoparticles from the vapor–solid grown mechanism by in situ temperature control. The obtained hierarchically-assembled ZnS nanostructures were characterized by various TEM technologies, verifying the crystal growth mechanism. Lastly, electron tomography and 3D printing enabled the nanoscale structures to visualize with centimeter scales. The 3D printing from randomly fabricated nanomaterials is rarely performed to date. The collaborating work could offer a better opportunity to fabricate advanced and sophisticated nanostructures.

## Introduction

Nanomaterials are of intense interest due to their enhanced surface properties, thus the nanomaterials can be used for high-performance energy storage and redox reaction platform, in vivo targeting drug delivery, additives for mechanical strength enhancement, and plasmonic light direction controller^1–7^. However, the manmade nanostructures preparation requires intricate fabrication steps to control the overall shape and precise position control of the nanomaterials on the targeting substrate. Correspondingly, there is an escalating need for novel visualization tools, which address rapid progress in developing various manmade nanoscale matters nowadays^8–12^. For visualization, the intricate structural details of such nanoscale matters should be directly inspected and verified at length scales relevant to their intrinsic near-atomic dimensions. Simultaneously, the obtained structural information and knowledge must be readily translated at much larger scales where additional assisting characterization tools are unnecessary for clarification and understanding.

Many studies have been conducted to prepare nanostructures from various methods such as chemical vapor deposition (CVD), thermal evaporation, and thermo-solution methods^12–18^. Then different types of nanostructures have been developed in the shape of the nanowire, nanoribbon, nanosheets, and nanoparticles. Although nanostructural fabrication technologies have been drastically developed, the prepared nanomaterials tend to agglomerate amongst the nanoscale structures, attenuating the intrinsic performance of the nanomaterials^19–23^. Thus, heterogeneous structures, such as core/shell structure and surface modification, have been studied to maintain and further enhance the functionality of nanomaterials^16–18,24–27^. Various nanostructures and their fabrication methods are continuously evolving in advanced heterogeneous structures, the methods however require complicated and exquisite processes. Transmission electron microscopy (TEM) is a typical tool to characterize near-atomic scale structures, capturing two-dimensional (2D) projected nanomaterials. The measuring technology helps the nanomaterials inspection to be more closely to atomic scales, hence the use of the technology would be a complementary strategy to fabricate advanced nanostructures^28,29^.

Although the TEM measurement broadened the nanomaterial preparation technique, the conventional TEM measurement often needs very sophisticated sample preparation and instrument operations for accessing spatial areas of specific interest. Three-dimensional (3D) TEM tomography has emerged as a powerful characterization tool owing to its unique ability to access structural details of nanoscale entities from all angles, which can grab details overlooked with the conventional approach^30–32^. The tomography operates based on accumulating and reconstructing 2D TEM images from matters of interest that are systematically rotated at various tilt angles inside the TEM device^33–35^. While its advantage for visualizing sophisticated structural details unattainable by the conventional tools is obvious, such information that is an accumulation of individual 2D TEM images assigned at specific tilt angles still presents a gap with truly 3D panoramic views. Also, grabbing and accessing the true 3D structures of nanoscale entities is not quite straightforward with 3D TEM tomography solely, particularly for those not familiar with TEM crystallographic analysis.

Here we suggest a method to prepare a complex and heterogeneous nanostructure using an in situ synthesis. The nanowire-based core–shell structures have been suggested in various research fields, such as photovoltaic devices, biomedical implantable electrodes, energy harvesting active layers, thermoelectric additives, and light absorption and reflection studies^24,36–40^. First, a hierarchically-assembled nanostructure was fabricated, consisting of a zinc sulfide (ZnS) nanowire (~ 50 nm diameter) core, ZnS nanoparticles on the surface of the ZnS nanowire, and Ag_2_S nanoscale catalyst on the tip of the ZnS nanowire. ZnS-based nanostructures fabrication has been widely studied with different dimensions due to its wide direct bandgap^41–43^. This characteristic enables the ZnS-based nanostructures to be used as optoelectronic applications such as light emitting diodes (LEDs), electroluminescent devices (ELDs), versatile sensors, and infrared windows^44–49^. The hierarchically-assembled nanostructures were prepared from simple, in situ synthesis by metal–organic chemical vapor deposition (MOCVD). Since the vapored or liquefied Zn and S can be solidified in the different shapes of nanostructures with temperatures (VLS pathway), the Zn and S are assembled into nanowire under Ag catalyst and then nanoparticles on the surface of the nanowire by the temperature control from vaporized Zn and S (VS pathway). To our knowledge, this is the first demonstration to synthesize heterogeneous nanostructures by VLS then VS grown mechanism from in situ temperature control solely.

The assembled nanostructures were monitored by various TEM techniques then characterized by 3D TEM tomography. Further, the 3D TEM tomography was combined with 3D printing technology to visualize the complex nanostructures. We suggest a collaborating characterization approach that directly visualizes 3D structural details of nanoscale entities and translates them to a much larger length (> 10 cm^2^) where their direct inspection is possible without additional characterization efforts^50–52^. Furthermore, the successive nanostructure inspection by 3D TEM tomography and visualization by 3D printing promotes elaborate nanofabrication, resulting in the enhanced and modified performance of the products^53–55^. Although many studies have been reported to materialize sophisticated structure using 3D printing from intentionally coordinating work^56–58^, rare study has been performed to print nanostructures from randomly fabricated materials.

The results should open up a novel field that allows easy-to-fabricate heterogeneous nanostructures and investigates with high spatial resolution. This could ultimately have an impact on our understanding of the crystal structure growth mechanism that plays an important role in the final nanostructure properties.

## Results

### Hierarchically-assembled ZnS nanostructures preparation

**Figure 1:**
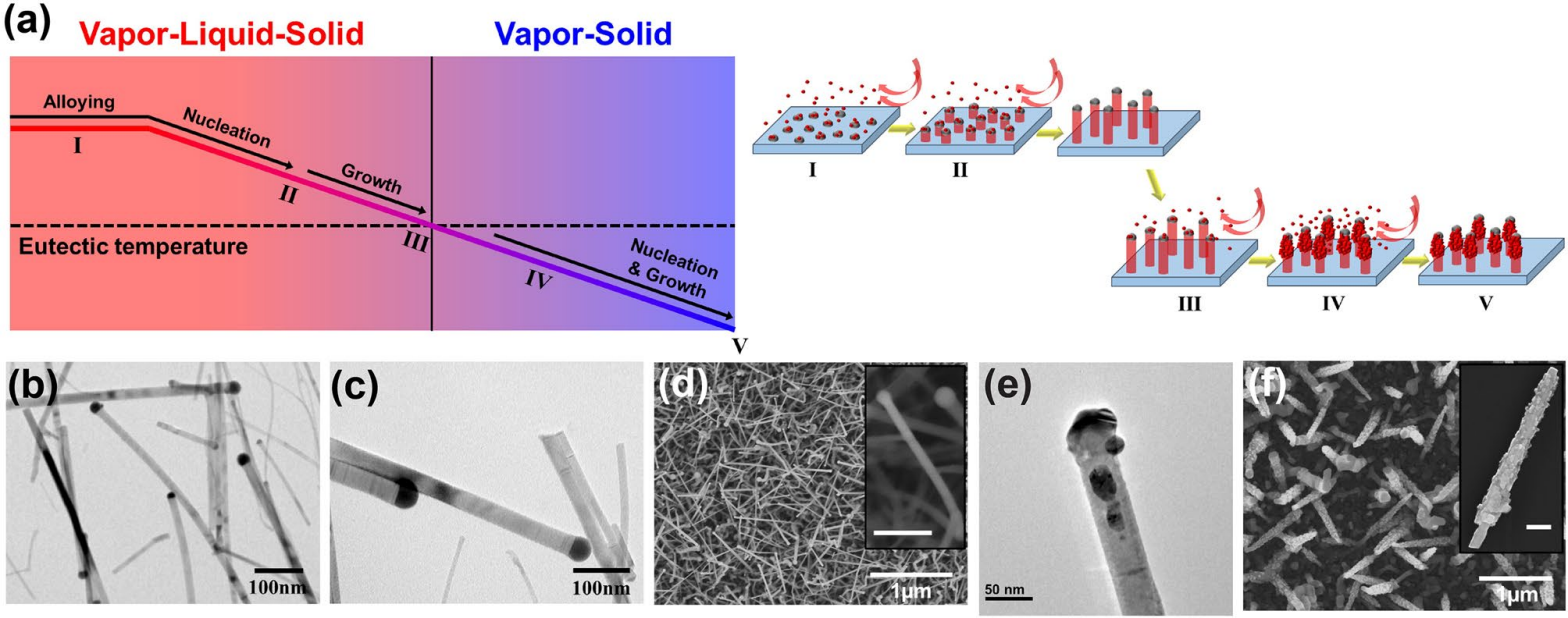
Hierarchically-assembled ZnS nanostructures preparation. (**a**) Schematic diagram of the hierarchically-assembled ZnS nanostructures growth mechanism. TEM images of ZnS nanowires with temperatures (II → III); (**b**) 800 °C and (**c**) 760 °C. (**d**) SEM image of ZnS nanowires at eutectic temperature (III). (**e**) Nanoparticles growth initiation (III → IV) confirmed by TEM image. (**f**) SEM image of ZnS nanostructures (V). Both insets in (**d**) and (**f**) displayed a single nanowire and nanostructure from high-magnification SEM images (scale bar: 100 nm).

The hierarchically-assembled ZnS nanostructures were grown here following five steps that can be seen in Fig. 1a: (I) The ZnS powder evaporates at 900 °C, separating into Zn (vapor) and S (vapor) precursors. The Ag thin film of 10 nm thickness coated on the Si wafer minimizes the surface energy and forms spherical Ag droplets of a liquid phase, while the gaseous Zn and S precursors are dissolved into the Ag droplets. The reaction between Ag (liquid) and S (vapor) is thermodynamically favored. Hence, Ag_2_S is formed first, and the continuously provided Zn and S are dissolved into the Ag_2_S droplet in the ZnS form^59,60^. (II) The temperature of the quartz tubing inside was decreased after 20 min when ZnS was sufficiently dissolved into the Ag_2_S droplet. Then the supersaturated ZnS is precipitated in a solid phase to form nanowires at the nucleation temperature. Then ZnS nanowire growth was monitored with temperatures at 800 °C (Fig. 1b) and 760 °C (Fig. 1c). The vapor–liquid-solid (VLS) growth process was suggested in the previous studies^61–65^. (III) The growth of the highly supersaturated ZnS nanowires (Fig. 1d) was stopped under a temperature of 700 °C, which is defined as “eutectic temperature” here. The grown ZnS nanowires clearly display the uniform length and diameter of the individual nanowire range of a few μm and a few tens of nm, respectively. (IV) The continuously provided Zn (vapor) and S (vapor) precursors were directly deposited on the nucleation in a solid mode to form ZnS nanoparticles around ZnS nanowires (Fig. 1e). This process was controlled by a vapor–solid (VS) growth mechanism. (V) The growth of the nanoparticles was automatically terminated when the temperature was cooled to room temperature.

The hierarchically-assembled ZnS nanostructures display that each ZnS nanowire was decorated by considerable amounts of nanoparticles with size < 10 nm, which generate the thickness of 50–100 nm (nanoscale) and the length of ~ 1 μm, respectively (Fig. 1f). XRD measurement corroborated the crystal structure of the as-obtained ZnS nanostructures, corresponding to hexagonal 2H wurtzite phase ZnS with lattice constants of a = b = 3.836 Å and c = 6.277 Å, which is quite different from those obtained from ZnS nanowire (Fig. S2).

### Hierarchically-assembled ZnS nanostructures characterization

**Figure 2:**
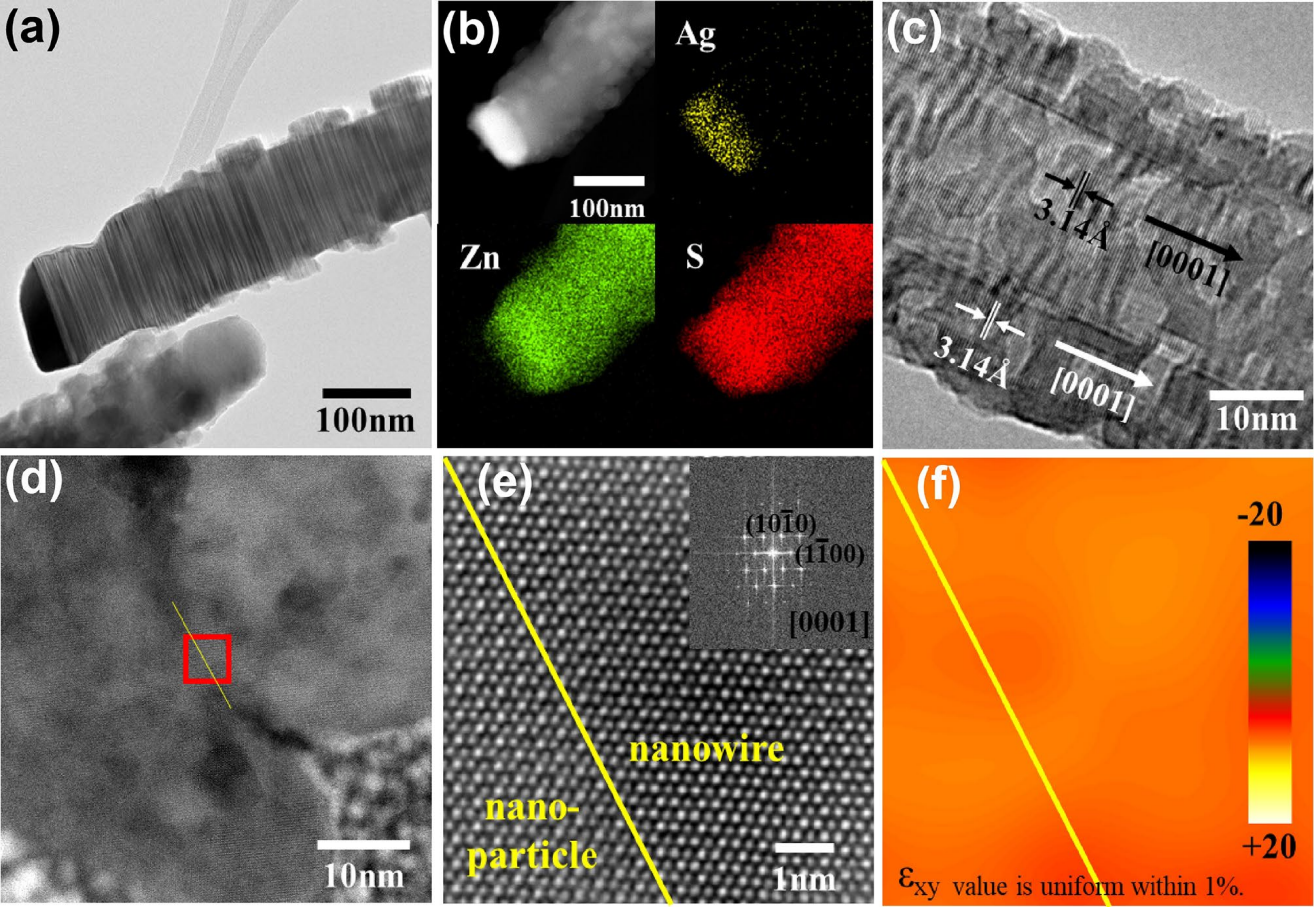
Structural analysis of the hierarchically-assembled ZnS nanostructures. (**a**) A BF TEM image of hierarchically-assembled ZnS nanostructure. (**b**) HR ADF-STEM images of the nanostructure and EDS element maps (yellow: Ag, green: Zn, red: S). (**c**) HR-TEM image of a single ZnS nanostructure showing the c-axis growth and single-crystalline nature. (**d**) BF TEM and (**e**) HR ADF-STEM images obtained from a cross-section of hierarchically-assembled ZnS nanostructures (inset: corresponding FFT pattern). (**f**) The strain field of (**e**) that was calculated by using GPA.

Various TEM studies demonstrated the ability to inspect a detailed morphology of the complex and random nanostructures. A typical bright field (BF) TEM image of the hierarchically-assembled ZnS nanostructure revealed distinctly irregular nanoparticles attached to the surface of the ZnS nanowire (Fig. 2a, low-magnification images in Fig. S3). The Ag_2_S catalyst was shown as a dark elliptical form on the end of the hierarchically-assembled ZnS nanostructure. TEM elemental mapping analysis of individual hierarchically-assembled ZnS nanostructure displayed that Zn and S elements are dominant in nanowire and nanoparticles (Fig. 2b). Ag signals are strongly detected at the tip of the nanostructure with the dense S element signals. The EDS mapping results support the finding that the ZnS nanowire was grown from the VLS method using the Ag_2_S catalyst then ZnS nanoparticles were grown around the nanowire from the VS method (Fig. S4).

The hierarchically-assembled ZnS nanostructure was further inspected using the high-resolution TEM (HR-TEM) images, examining the detailed crystal structures of both ZnS nanowire and nanoparticles (Fig. 2c); this shows highly crystallized lattice fringes for both the ZnS nanowire and nanoparticles. The lattice image determined the interplanar spacing of 0.314 nm perpendicular to the ZnS nanowire axis that was exactly the [0001] lattice plane. The ZnS growth was in the [0001] direction of the wurtzite ZnS. The ZnS nanoparticles also showed a [0001] lattice plane with an interplanar spacing of 0.314 nm, and ZnS growth was in the [0001] direction.

The FIB technique performed cross-sectioning of the ZnS nanostructures. High-resolution annular dark-field STEM (HR ADF-STEM) image of the specific area marked with a red square (Fig. 2d) was well-matched with hexagonal ZnS regarding Z-contrast between Zn and S (Fig. 2e). The right inset displayed the fast fourier transformation (FFT) with [0001] zone axis. The diffraction spots are indexed to wurtzite ZnS, confirming that the hierarchically-assembled ZnS nanostructures are single crystalline. The strain field between nanowire and nanoparticle by GPA tool (Fig. 2f) displayed the overlapped strain mapping for ε_xy_, and the mapping data clearly showed that strain is not induced (less than 1%) between nanowire and nanoparticle.

### Hierarchically-assembled ZnS nanostructures visualization

**Figure 3:**
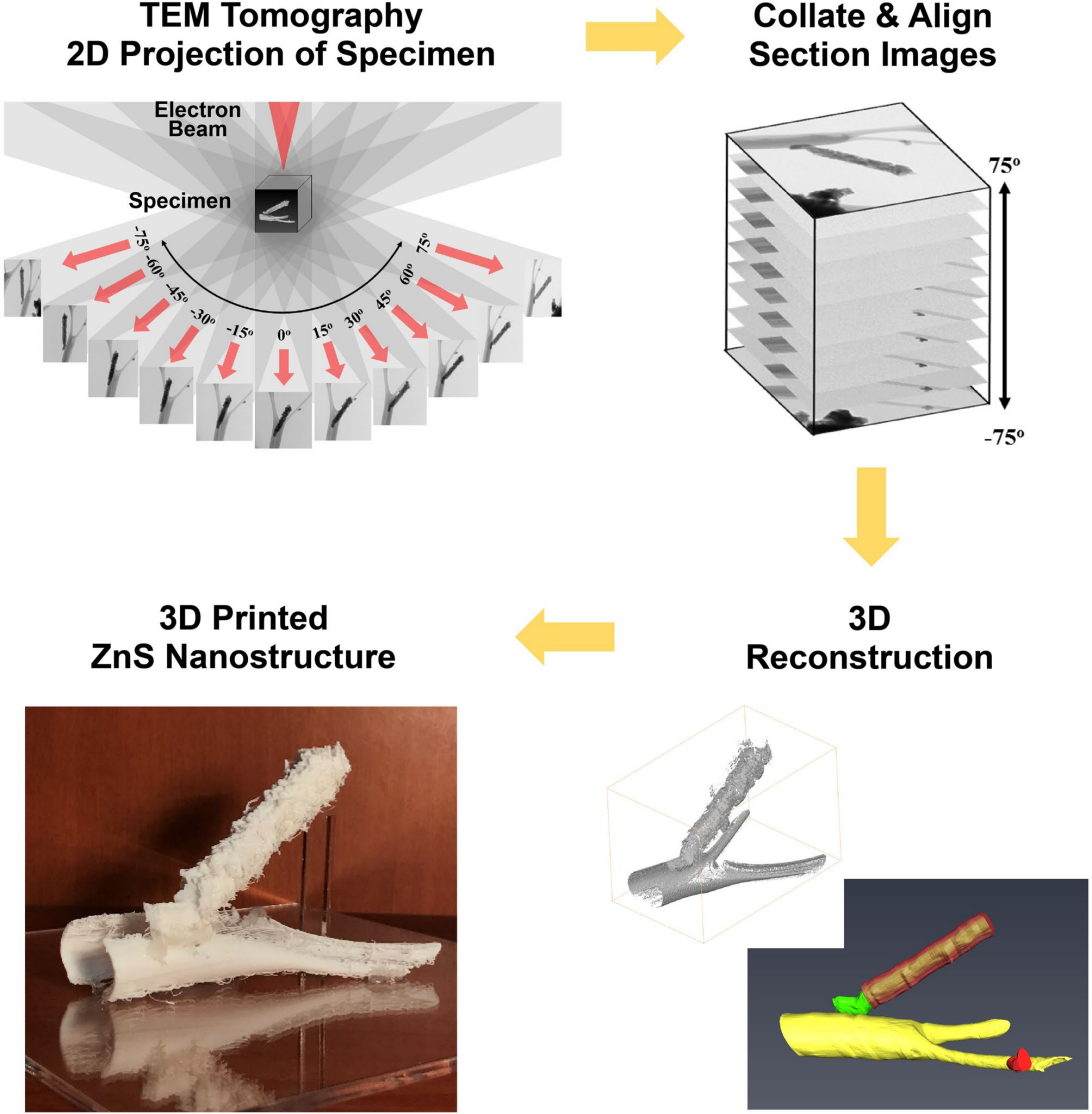
Schematic illustration of TEM tomography data acquisition, 3D reconstruction, and 3D printing result.

3D tomography analysis of the hierarchically-assembled ZnS nanostructure with a lacey carbon grid was performed inside the TEM, obtaining better insight into the specific nanostructure. Several sets of tilted BF TEM images were collected at tilt intervals of multi-degrees from − 75° to 75° using the rotation holder (rotation video can be seen in Movie S1). These electronic tomographs reconstructed 3D objects from 2D projection images taken at different viewing angles and are made into 3D objects from combined 2D images through the following four processes (Fig. 3): (1) Series of 2D images acquired of the hierarchically-assembled ZnS nanostructure at the different viewing angles. (2) 2D projected images combined into an image stack ordered by viewing angle. (3) Tilt series is aligned, and a reconstruction algorithm is applied to produce a 3D reconstruction of the nanostructure (Movie S2 for tomography and Movie S3 for 3D visualization). (4) The hierarchically-assembled ZnS nanostructure was output using 3D printing.

**Figure 4:**
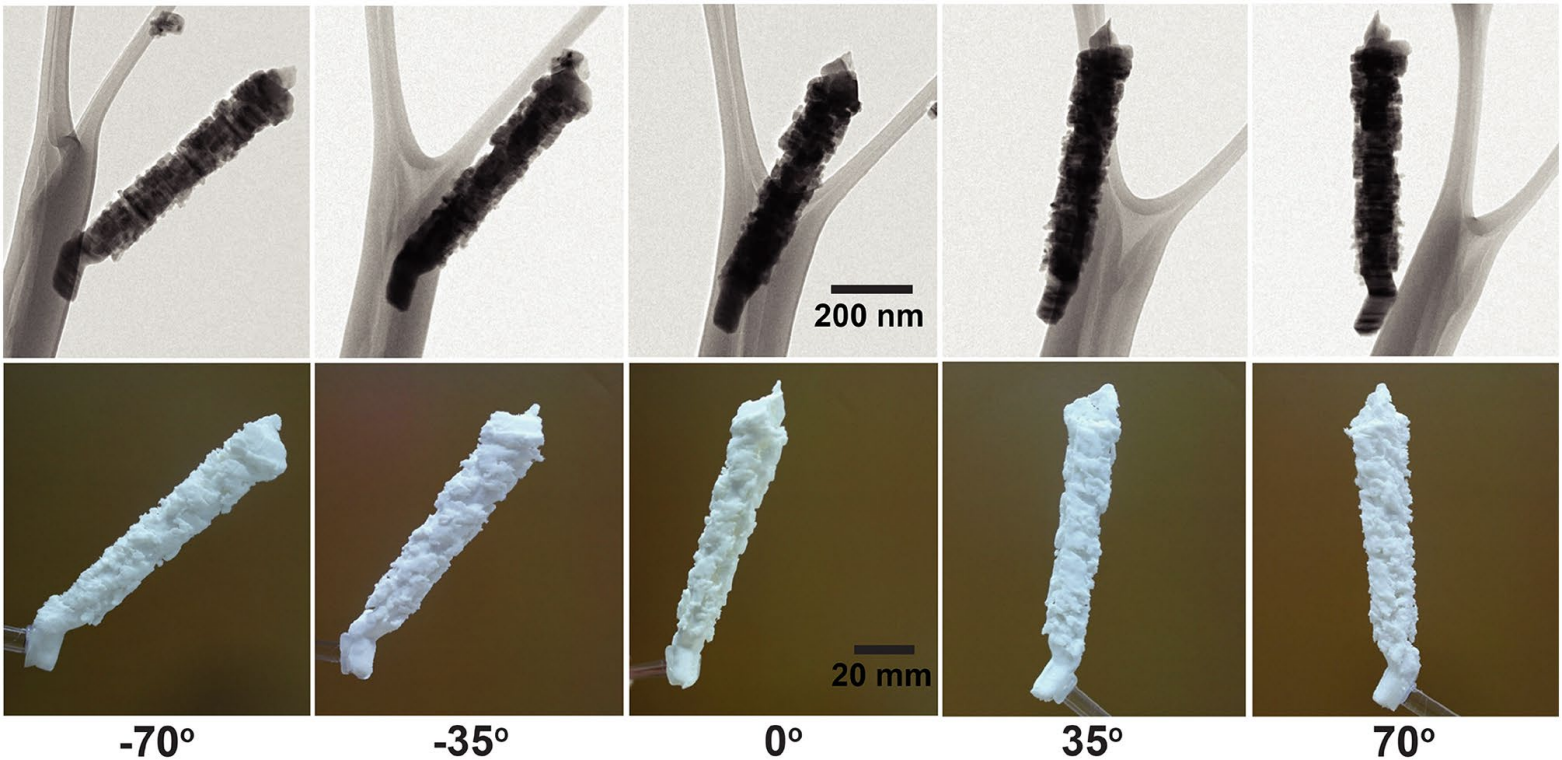
Various tilt angles BF images of a tomographic series (top) and photographs of the 3D printed (magnified over 10^5^ scales) hierarchically-assembled ZnS nanostructures (bottom).

**Figure 5:**
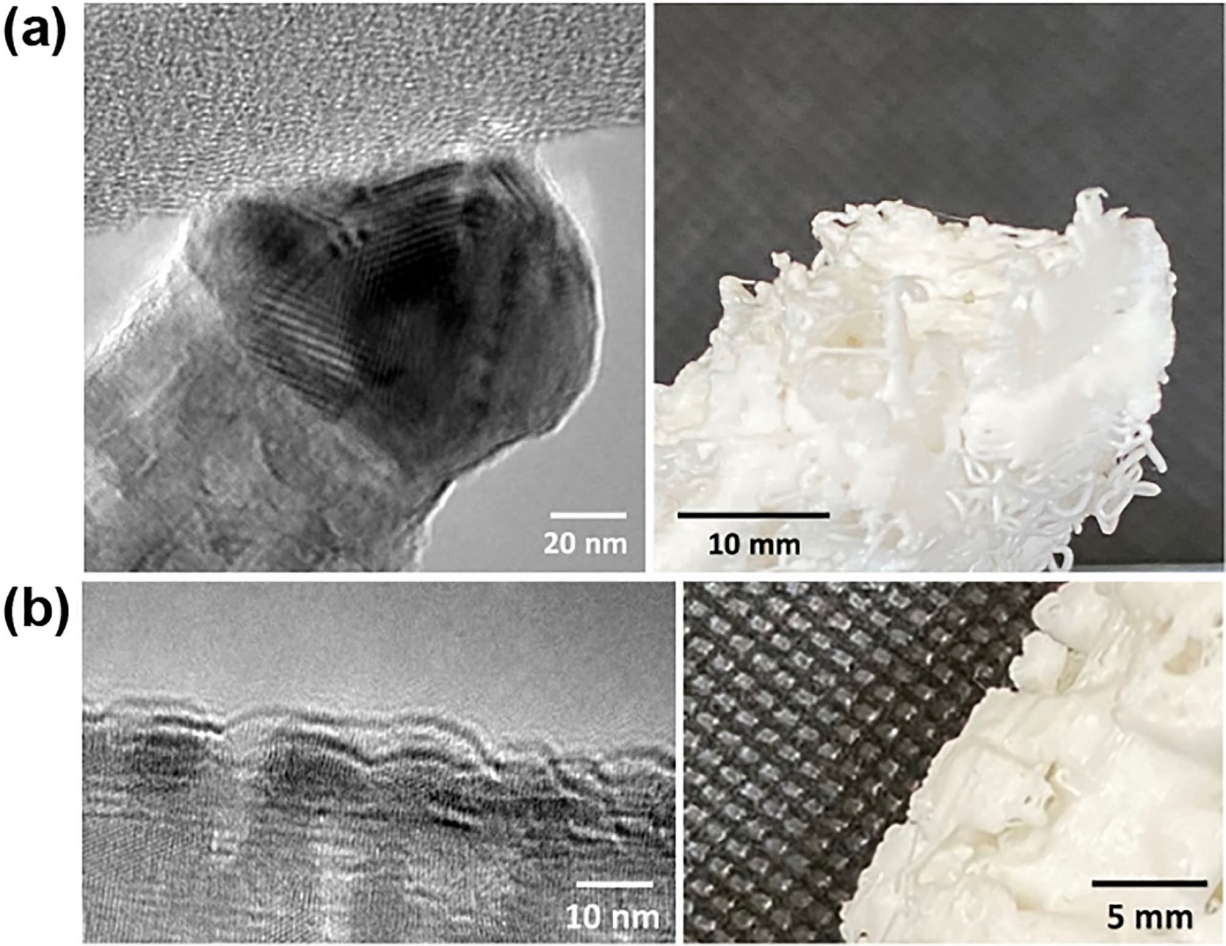
Magnified optical images (right) to confirm the visualization from the nanoscale measurement (left); (**a**) tip and (**b**) surface of the hierarchically-assembled ZnS nanostructure.

Five representative BF images of the hierarchically-assembled ZnS nanostructures tilted at − 70°, − 35°, 0°, 35°, and 70° were displayed in Fig. 4 top. The shape and location of the nanoparticles are revealed in the reconstructed volume of 3D printed in Fig. 4 bottom. The 3D printed nanostructures provided a length scale that is 10^5^ magnified than the pristine nanostructures. A crucial aspect of electron tomography is segmenting the features of interest from the 3D reconstructions and quantitatively describing their properties. Lastly, visualization accuracy was confirmed by the comparison between TEM measured images and magnified optical images (Fig. 5). The comparison demonstrated that the visualization successfully reproduced the nanoscale measurement to centimeter scale artifact, enabling the complicated nanostructures to be easily observed then ultimately modified by the visualization feedback.

## Discussion

The hierarchically-assembled ZnS nanostructures were prepared by VLS and VS growing methods. Vaporized Zn and S dissolved in liquefied Ag_2_S catalyst then formed ZnS nanowire under saturated temperature. The vaporized Zn and S were directly attached around the ZnS nanowire under eutectic temperature (700 °C) and formed ZnS nanoparticles. The in situ method controlled by temperature solely should open up the numerous possibilities of fabricating heterogeneous hierarchically-assembled nanostructures. Various TEM measurements demonstrated the hierarchically-assembled ZnS nanostructures and crystal growth direction directly. In addition, 3D tomography helped the nanostructures to reveal their specific features. Lastly, the nanostructures were visualized by the 3D printing technique on large scales (> 10 cm^2^). This study would enable the nanostructure fabrication to be more precise, resulting in high-performance nanomaterial products.

## Methods

### ZnS nanostructures preparation setup

The hierarchically-assembled ZnS nanostructures were prepared in horizontal quartz tubing with a gold furnace system (see Fig. S1). First, a Si (100) wafer (1.0 cm × 3.0 cm) was cleaned in acetone and sonicated for 10 min. Then Ag film (10 nm thickness) as a catalyst was deposited on the cleaned Si wafer for 10 s via ion sputtering. Next, 0.1 mg zinc diethyldithiocarbamate (97%, ZnS powder, Sigma-Aldrich) was poured into an alumina boat placed upstream of the quartz tubing. The Ag coated Si wafer was located downstream of the quartz tubing. After reducing the pressure of the quartz tubing inside to 0.1 torr, a mass flow controller (MFC) was used to set a 100 sccm (standard cubic centimeter per minute) flow of Ar gas (99.999% purity). The pressure of the quartz tubing inside was maintained at 5.0 torr using the pressure control system. When the gold furnace temperature reached 900 °C with a rate of 1 °C/s, the quartz tubing, including the alumina boat upstream and the Ag coated Si wafer downstream, moved inside the furnace.

### Structural characterizations

The morphologies and microstructures of the as-synthesized products were systematically characterized. The crystalline structure characteristics and the morphology of the synthesized hierarchically assembled ZnS nanostructure were investigated using an X-ray diffractometer (XRD, Bruker D8 advance), field-emission scanning electron microscopy (FE-SEM, Hitachi-8230), and Cs-corrected transmission electron microscopy (Cs-TEM JEOL ARM200F) operating 200 kV. The chemical composition of the hierarchically assembled ZnS nanostructure was analyzed using an energy dispersive X-ray spectrometer (EDS, Oxford Aztec 80 T) attached to the Cs-TEM. A focused ion beam (FIB, FEI NOVA200) was used to observe the cross-section of the hierarchically assembled ZnS nanostructure. A geometric phase analysis (GPA) script installed on a Gatan Digital Micrograph was used to calculate the strain generated at the interface between the ZnS nanowire and the ZnS nanoparticle.

### TEM tomography and 3D printing

TEM tomography was performed using the automated tilt-series acquisition software TEMography on a Cs-TEM operated at 200 kV. The first step in TEM tomography is recording TEM images while tilting the sample over a wide range of angles in small increments. For the ZnS nanowires coated with nanoparticles, JEOL rotation holder was employed to collect bright field (BF) images in a Cs-TEM. The tilt of the JEOL rotation is motor-driven from 0 to 360°. 160 BF images were collected for each reconstruction at 1° tilt intervals in a tilt range of − 80° to + 80°. Here lacey carbon grid, which was used as a template for TEM analysis, was included to visualize the whole nanostructures more sophisticatedly. Composer (TEMography.com) performs complete automatic acquiring of sequential tilted TEM images series essential for tomographical 3D image reconstruction. Algorithms corresponding to inherent problems, such as correction of shifted position when specimen tilted and maintenance of focus, are applied automatically. After data acquisition, all BF TEM images are aligned with respect to a common origin and tilt axis. In the next step, a 3D reconstruction of the imaged sample is computed using specialized algorithms such as filtered back-projection (FBP) and the simultaneous iterative reconstruction technique (SIRT). The 3D reconstructed result and Amira software (ThermoFischer Scientific) provide the basis to visualize the ZnS nanostructure morphology. The hierarchically-assembled ZnS nanostructure forms were fabricated by a 3D printing approach with the polylactic acid (PLA) filament. The used custom-made 3D printing system consisted of a fused-filament-fabrication (FFF) head with a 300 μm nozzle, a movable printing bed on a three-axis, and an optical monitoring module; the monitoring module consisted of an optical lens (10 ×) and a charge-coupled device (CCD) camera (Blackfly USB3 FLIR). The template of the hierarchically assembled ZnS nanostructure form was obtained with TEM tomography and exported as stereolithography (.stl) file into TEMography (Visualizer-kai, Japan). The 3D structures, which were formed in .stl format, were fabricated in a layer-by-layer 3D printing manner. After printing, the support part that maintained the shape of the printed structures during the printing process was removed.

## Supplementary Information

Supplementary Figures.

Supplementary Movie S1.

Supplementary Movie S2.

Supplementary Movie S3.

## Data Availability

All the data produced by this study are included in this published article.
