# Supplementary material for: In situ synthesis of hierarchically-assembled three-dimensional ZnS nanostructures and 3D printed visualization
Source: Sci Rep. 2022 Oct 10;12:16955. doi: 10.1038/s41598-022-21297-y (PMC9550785; doi:10.1038/s41598-022-21297-y)
Supplement: Supplementary file 1 — Supplementary Figures. [file 41598_2022_21297_MOESM1_ESM.docx]

**Supporting Information**

In situ Synthesis of Hierarchically-Assembled Three-Dimensional ZnS Nanostructures and 3D printed Visualization

Taehwan Lim^1^, Seung Kwon Seol^2,3^, Hyo-Jeong Kim^4^, Yang Hoon Huh^4^, Yeonwoong Jung^5,6,7*^, Hee-Suk Chung^8,*^, Jung Han Kim^9,*^

^1^Advanced Textile R&D Department, Korea Institute of Industrial Technology, Ansan, Gyeonggi-do 15588, South Korea

^2^Smart 3D Printing Research Team, Korea Electrotechnology Research Institute, Changwon 51543, South Korea

^3^Electrical Functionality Material Engineering, University of Science and Technology (UST), Changwon 51543, South Korea

^4^Electron Microscopy Research Center, Korea Basic Science Institute, Ochang 28119, South Korea

5NanoScience Technology Center, University of Central Florida, Orlando, Florida 32826, USA

^6^Department of Electrical and Computer Engineering, University of Central Florida, Orlando, Florida 32816, USA

^7^Department of Materials Science and Engineering, University of Central Florida, Orlando, Florida 32826, USA

^8^Analytical Research Division, Korea Basic Science Institute, Jeonju, Jeollabuk-do 54907, South Korea

^9^Department of Materials Science and Engineering, Dong-A University, Busan 49315, South Korea

Contents

Figures S1-S4

Movies S1-S3

*Authors to whom correspondence should be addressed: [YeonWoong.Jung@ucf.edu](mailto:YeonWoong.Jung@ucf.edu), [hschung13@kbsi.re.kr](mailto:hschung13@kbsi.re.kr), [junghankim@dau.ac.kr](mailto:junghankim@dau.ac.kr)

**
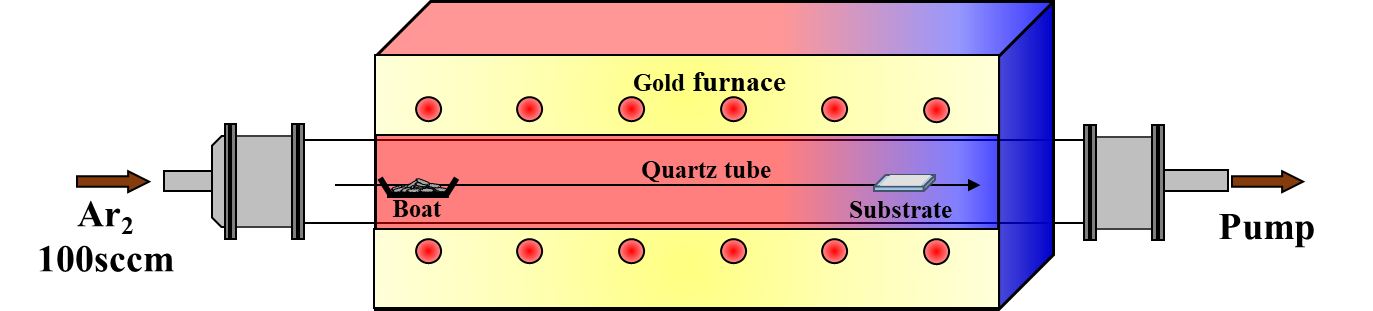
**

**Figure S1.** Schematic illustration of the horizontal quartz tubing with gold furnace system setup.


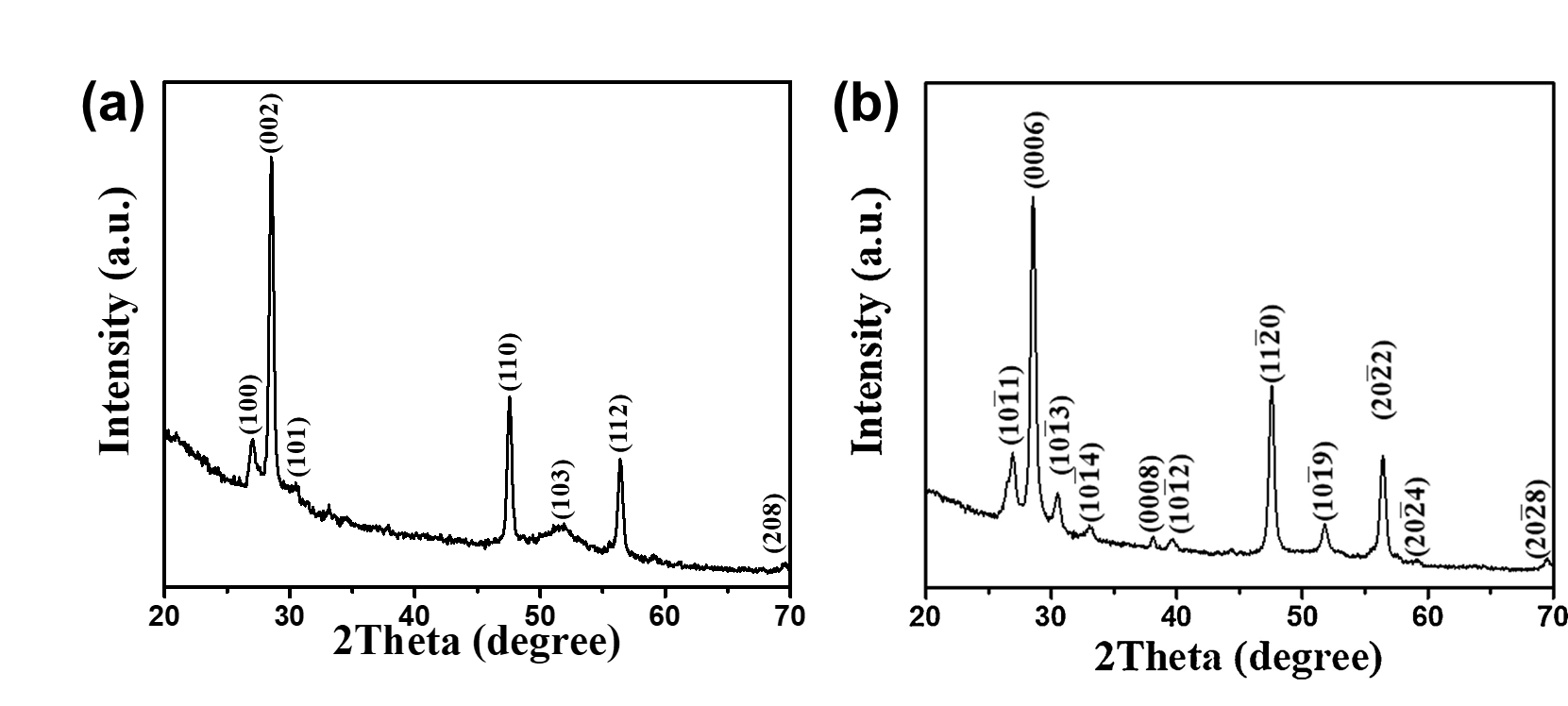


**Figure S2.** XRD pattern of (a) ZnS nanostructures and (b) ZnS nanowire. “Reprinted from Appl. Surf. Sci., 436, J.H. Kim, J.G. Kim, J. Song, T. Bae, K. Kim, Y. Lee, Y. Pang, K.H. Oh, H. Chung, Investigation of the growth and in situ heating transmission electron microscopy analysis of Ag_2_S-catalyzed ZnS nanowires, 556-561, 2018, with permission from Elsevier.” The peaks of the ZnS nanostructures are identified with a wurtzite 2H ZnS structure with lattice constants a=b=3.836 Å and c=6.277 Å. While the peaks of the ZnS nanowire are verified with the hexagonal wurtzite phase of ZnS with lattice constants of a=b=3.823 Å and c=18.743 Å.


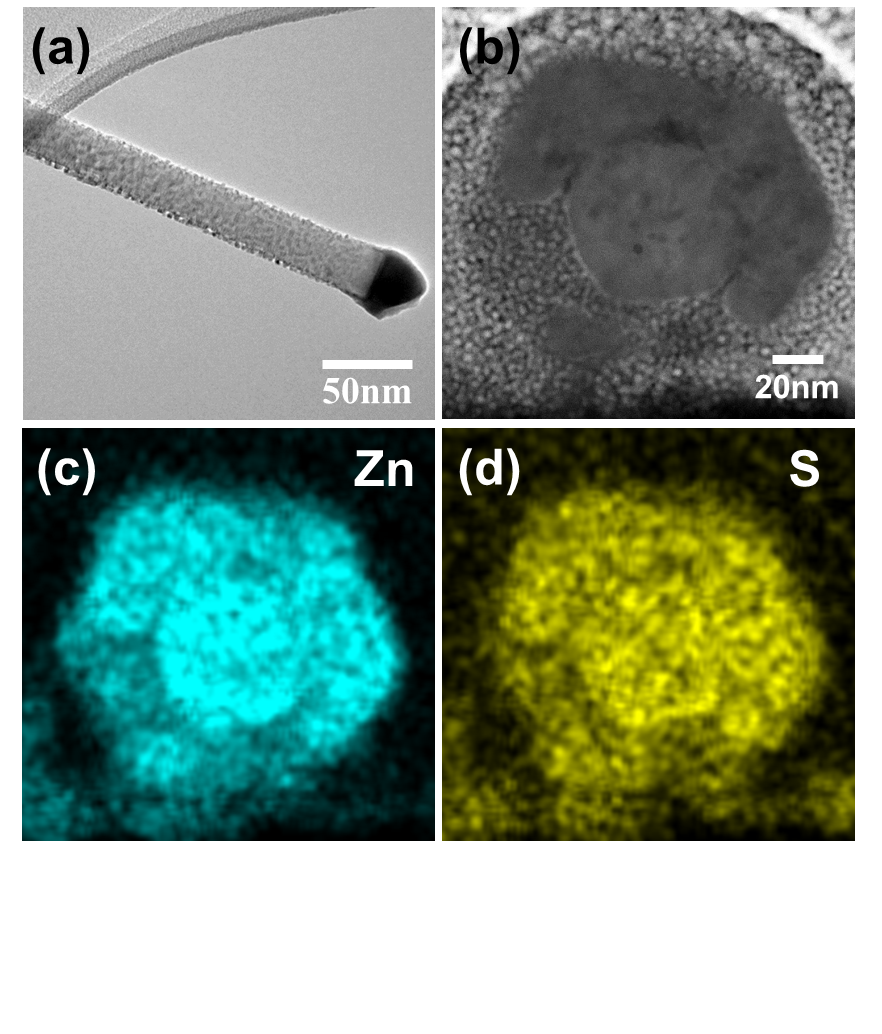


**Figure S3.** TEM images of hierarchically-assembled ZnS nanostructure: (a) side and (b) cross-sectioned. (c) and (d) indicated elemental analysis results (Zn and S) of the nanostructure cross-section.


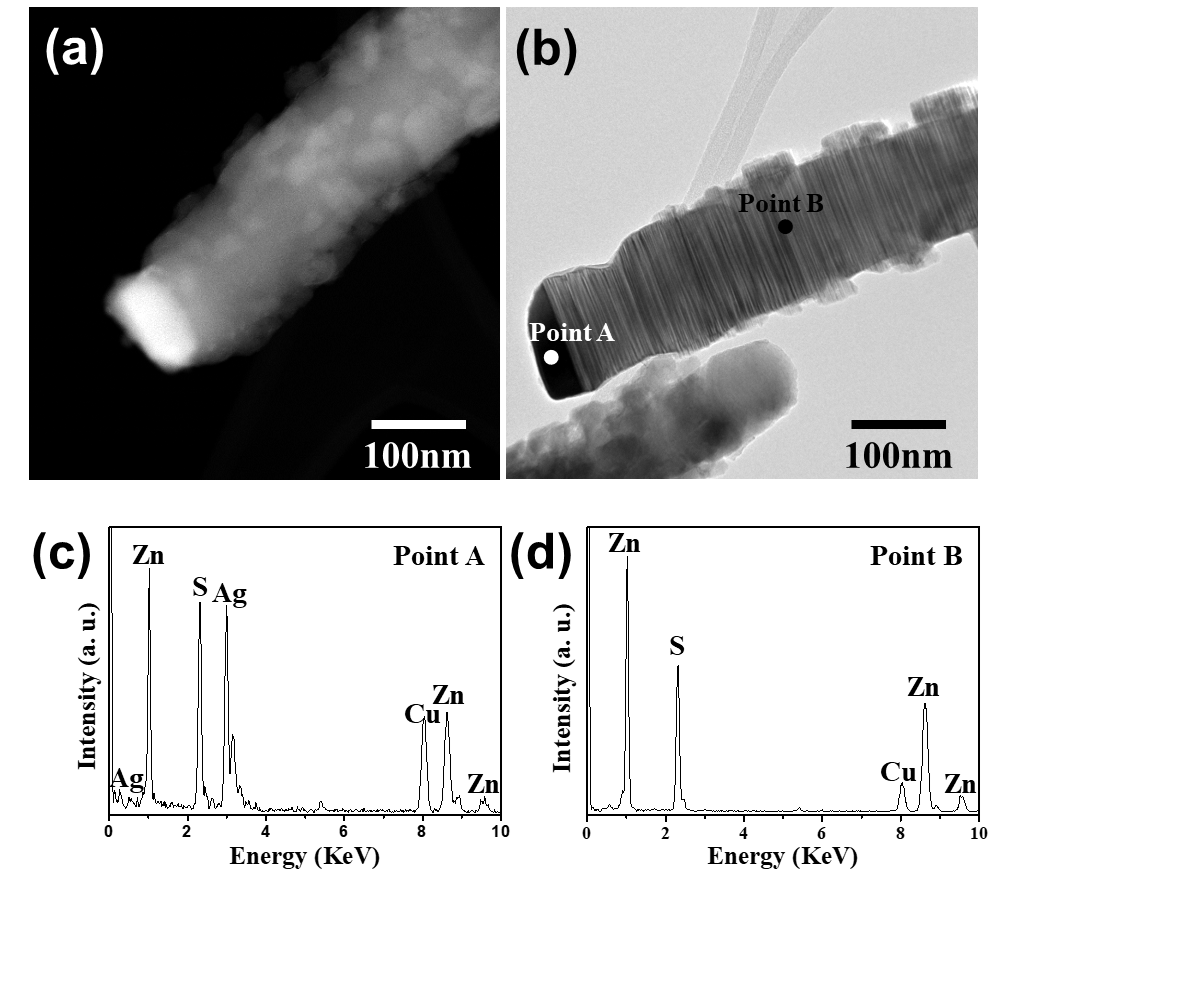


**Figure S4.** (a) SEM and (B) TEM images of the hierarchically-assembled ZnS nanostructures. (c) and (d) indicated elemental analysis results (points A and B, noted in **Figure 4b**).

**Movie S1.** Rotation image of the ZnS nanostructures prepared by TEM measurement.

**Movie S2.** Rotation TEM tomography of the ZnS nanostructures.

**Movie S3.** Reconstructed ZnS nanostructures from visualization program (Amira software).
